# Improving Medication Safety Through Medication Reconciliation in Pediatric Neurology: Clinical Pharmacist Recommendations and Physician Uptake in a 13-Week Study

**DOI:** 10.3390/children12050625

**Published:** 2025-05-12

**Authors:** Margherita Zennaro, Anna Trotter, Daniele Mengato, Laura Camuffo, Claudio Ancona, Irene Toldo, Maria Cecilia Giron, Maria Federica Pelizza, Margherita Nosadini, Giorgio Perilongo, Stefano Sartori, Francesca Venturini

**Affiliations:** 1 Hospital Pharmacy Department, Azienda Ospedale-Università Padova, Via Giustiniani, 35128 Padua, Italy; margherita.zennaro@aopd.veneto.it (M.Z.); laura.camuffo@aopd.veneto.it (L.C.); francesca.venturini@aopd.veneto.it (F.V.); 2 Department of Pharmaceutical and Pharmacological Sciences, University of Padua, 35131 Padua, Italy; anna.trotter@studenti.unipd.it (A.T.); cecilia.giron@unipd.it (M.C.G.); 3 Paediatric Neurology and Neurophysiology Unit, Department of Women’s and Children’s Health, University Hospital of Padua, 35128 Padua, Italy; claudio.ancona@aopd.veneto.it (C.A.); irene.toldo@unipd.it (I.T.); mariafederica.pelizza@aopd.veneto.it (M.F.P.); margherita.nosadini@aopd.veneto.it (M.N.); giorgio.perilongo@unipd.it (G.P.); stefano.sartori@unipd.it (S.S.)

**Keywords:** clinical pharmacy, pediatric care, medication reconciliation, interprofessional collaboration, drug therapy optimization

## Abstract

**Background/Objectives:** Clinical pharmacy plays a crucial role in optimizing medication use, particularly in pediatric settings where drug therapy can be complex and understudied. This study aims to assess the impact of clinical pharmacists in the Pediatric Neurology and Neurophysiology Unit of the Padova University Hospital, focusing on physician acceptance of pharmacist suggestions and the types of advice most frequently followed. **Methods:** A retrospective observational study was conducted over 13 weeks to describe the implementation phase of clinical pharmacists’ involvement in medication reconciliation in this setting. The study consisted of three steps. The study utilized a cluster model to categorize pharmacist suggestions and to evaluate physician acceptance rates. **Results:** The study included 57 hospitalized pediatric patients (53% male) with a median age of 3 years (IQR: 1–10.25). A total of 138 recommendations were shared, with an overall acceptance rate of 42%. Medication errors accounted for the largest cluster of suggestions (45%), though only 32% were accepted. Among the most frequently shared categories of suggestions, pharmaceutical form optimization (A) and drug supply (E) exhibited higher acceptance rates by clinicians (64% and 42%, respectively). The acceptance rate increased over time, peaking at 100% during weeks 7 and 11, correlating positively with enhanced collaboration between pharmacists and clinicians (R^2^ = 0.59). **Conclusions:** This study highlights the importance of clinical pharmacists in pediatric care, particularly in improving medication management through targeted interventions. The findings suggest that integrating clinical pharmacists into multidisciplinary teams can enhance patient care quality by fostering collaboration and trust among healthcare professionals.

## 1. Introduction

Clinical pharmacy constitutes both a professional discipline and a research field dedicated to optimizing pharmacotherapy, with the goal of enhancing patient-centered outcomes and advancing public health [1,2,3]. From this perspective, medication review and reconciliation are essential tools for tailoring drug therapy, particularly in complex or understudied settings like pediatric patients.

The limited availability of efficacy and safety data in pediatric populations, combined with the frequent off-label use of medications and the need for dosage form adaptations to ensure adherence, renders children a particularly complex group in drug therapy [2,4,5].

Moreover, children exhibit lower precision in symptom reporting compared to adults, with verbal reliability influenced by factors such as age, previous experiences with medications and illnesses, self-esteem, and resilience [6,7].

Although most research on therapeutic errors focuses on adult populations, children are up to three times more likely to experience adverse drug events and reactions resulting from such errors [8].

In this context, the clinical pharmacist plays a pivotal role in optimizing pharmacological management. A study conducted at Hacettepe University, Ankara, evaluated the impact of clinical pharmacist-led intervention for drug-related problems (DRPs) in neonatal intensive care units. The results revealed a significantly lower incidence of medication errors in the intervention group (35%), in which clinical pharmacists were involved, compared to the control group (53%), along with improved accuracy in drug prescribing and administration. However, the incidence of adverse drug reactions—most commonly electrolyte imbalances and nephrotoxicity—did not differ significantly between groups [9].

These findings underscore the need for personalized and targeted approaches in pediatric drug use to enhance safety and improve care quality. The clinical pharmacist plays a critical role in advancing the care of hospitalized pediatric patients by contributing to the development and implementation of individualized therapeutic strategies. Effective integration of this role necessitates strong interdisciplinary collaboration, often within the core care team, to facilitate shared decision-making and optimize patient outcomes [10].

Physicians’ acceptance of clinical pharmacist recommendations has been widely explored in the literature, with findings showing considerable variability. Although some studies report high acceptance rates, others highlight fluctuations influenced by clinical settings and the nature of interprofessional relationships. Key factors affecting acceptance include the level of trust in pharmacists, the clarity and clinical relevance of the recommendations, and the robustness of the supporting evidence [11].

This study aims to comprehensively assess the role of clinical pharmacists in the Pediatric Neurology and Neurophysiology Unit, with a particular focus on physician acceptance of pharmacists’ suggestions and the types of recommendations most frequently implemented over a 13-week period. The Pediatric Neurology and Neurophysiology Unit is a bed academic unit specializing in the management of epilepsy, neuromuscular disorders, pain syndromes, and neurobehavioral conditions. Most patients were treated for epilepsy (approximately 70%), with others admitted for muscle tone abnormalities, pain management, and behavioral disorders. Given that the majority of patients in this unit are treated for epilepsy, this setting presents a critical area for clinical pharmacy interventions, as antiepileptic therapies require careful monitoring to ensure both efficacy and safety.

## 2. Materials and Methods

### 2.1. Study Design

**Figure 1:**
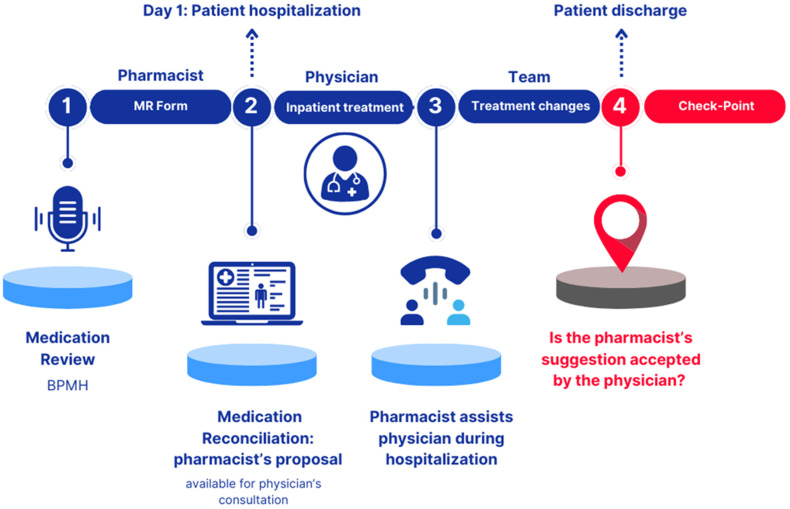
Graphical representation of the study design, depicting Phases 1, 2, and 3. BPMH: Best Possible Medication History. MR Form: Medication Review Form.

A retrospective observational academic study was conducted over a 13-week period, from June to September 2024, in the Pediatric Neurology and Neurophysiology Unit of the Padova University Hospital, Northern Italy. The study presents the implementation phase of a medication reconciliation service conducted by clinical pharmacists and developed across three distinct steps, as illustrated in Figure 1.

#### 2.1.1. Phase 1 “Pharmacist: Medication Review Form”

Phase 1 was conducted in a pre-hospitalization setting and involved collecting the Best Possible Medication History (BPMH) for each patient, followed by the completion of the Medication Review (MR) Form. The BPMH was gathered using two separate sources of information:

- A phone interview with the patient’s parent/caregiver.
- A review of clinical documentation, available through the electronic medical record.

An in-depth analysis of each patient’s therapy was subsequently performed. To facilitate this, the following resources were utilized:

1. Evidence-based point-of-care medical resources, including UpToDate (UpToDate Inc., Waltham, MA, USA) and Merative Micromedex (Merative LP, Ann Arbor, MI, USA) [12,13].
2. Summary of Product Characteristics (SmPC), Instructions for Use (IFU), or product sheet databases for medicinal products, medical devices, or non-medicinal products, respectively. The completed MR Form was signed by the pharmacist and uploaded into the patient’s electronic medical record. The MR Form collected the following information: patient’s anonymized name, surname, and date of birth (for data analysis); allergies and/or intolerances; recent adverse drug reactions; recently discontinued therapies; meal administration times and feeding route; details of in-use medicinal products, including name, dosage, posology, pharmaceutical form, posology, and route/method of administration; non-medicinal products (e.g., medical devices, homeopathic remedies, supplements); drug–drug, food–drug, and drug–other non-medicinal product contraindications or major interactions; and any other relevant information (e.g., drug handling, supply, or administration issues). The key areas of focus for the reconciliation proposal included pediatric dosages, formulation stability data, and strategies to improve drug administration, either orally or via Percutaneous Endoscopic Gastrostomy (PEG) or Nasogastric Tube (NGT).

#### 2.1.2. Phase 2 “Physician: Inpatient Treatment”

The MR Form, generated in Phase 1, was available for consultation by physicians and served as a medication reconciliation proposal. In this phase, the pharmacist’s recommendations were either confirmed or rejected by the physicians.

#### 2.1.3. Phase 3 “Team: Treatment Changes”

During patient hospitalization, the clinical pharmacist provided support to physicians regarding drug administration, supply, and handling. A dedicated telephone line was established between doctors and the clinical pharmacist, who was also actively involved in the Unit’s weekly meetings. During this phase, the pharmacist’s recommendations could still be accepted or rejected by the physicians.

#### 2.1.4. Checkpoint

After the patient’s discharge, a follow-up was conducted to determine whether the pharmacist’s recommendations regarding both in-hospital and post-discharge therapy were accepted by the prescribing physician. Additionally, the specific suggestions that were accepted were identified. This checkpoint represents the primary objective of the study.

### 2.2. Cluster Model

Following a thorough evaluation of the PCNE Classification for Drug-Related Problems V9.1 [14], a classification system for the potential recommendations provided by clinical pharmacists was developed.

**Figure 2:**
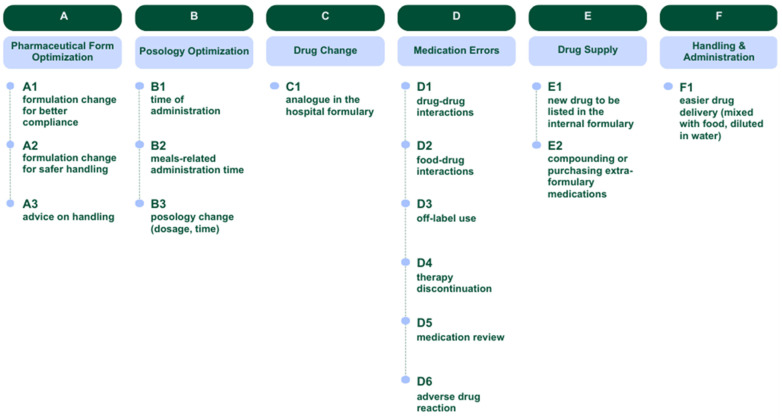
Cluster model: types of recommendations provided by the clinical pharmacist.

Six clusters of suggestions were identified: pharmaceutical form optimization (A), posology optimization (B), drug change (C), medication errors (D), drug supply (E), and handling and administration (F). Additionally, each recommendation was assigned an alphanumeric code (letter + digit), as illustrated in Figure 2.

### 2.3. Inclusion and Exclusion Criteria

Patient selection was based on the following criteria:

1. Patients with a scheduled hospitalization in the Pediatric Neurology and Neurophysiology Unit.
2. Patients prescribed at least two different concomitant daily medications, regardless of dosing frequency, were included.
3. Hospitalized patients with a high care burden (e.g., requiring multiple daily administrations and/or manipulations of the pharmaceutical form).
4. Patients capable of providing informed consent or whose guardians provided consent on their behalf.

Lack of a scheduled hospitalization or absence of pharmacological therapy were not considered exclusion criteria.

Both inclusion and exclusion criteria are explicitly stated in this section for clarity.

### 2.4. Outcome Measures

The primary outcome was the degree of physician acceptance of the medication review activities performed by the clinical pharmacist.

Secondary outcomes included the types of recommendations most commonly accepted by physicians and the correlation between time to intervention and the number of accepted recommendations. Time to intervention was measured in weeks from baseline (day 0).

The percentages of physician-accepted suggestions out of the total number of pharmacist-supplied recommendations were calculated for each patient, categorized by type of suggestion and over time. These percentages represented the primary and secondary endpoints.

### 2.5. Statistical Analysis

Continuous normally and non-normally distributed variables were reported as medians (interquartile range, IQR), whereas categorical variables were expressed as absolute values and percentages.

For correlation analysis, a positive correlation was considered when R^2^ > 0.50.

Data analysis was performed using Jamovi software (Jamovi Desktop, version 2.6.22) [15].

### 2.6. Ethics Approval

The study was conducted in compliance with Good Clinical Practice (GCP) standards as outlined in the ICH guideline for Good Clinical Practice E6(R2) Step 5 [16].

Informed consent for all patients enrolled in the study was obtained from their legal guardians [17]. Formal approval was granted by the local Ethics Committee (CET-ACEV) on 7 March 2024 (code: CET-ACEV: 469n/AO/24).

## 3. Results

### 3.1. Baseline Characteristics of the Cohort

A total of 63 patients were initially enrolled in the study, with a clinical pharmacist completing an MR Form for each participant. However, 6 patients scheduled for hospitalization were not admitted, leading to their exclusion from the analysis, resulting in a final cohort of 57 patients.

Of the 57 patients, 30 (53%) were male. The median age of the patients was 3 years (IQR: 1.00–10.25), with 19% being younger than one year and a similar proportion being adolescents. Regarding their pharmacological therapy, 16% of the patients were prescribed at least 5 medications per day, resulting in a median daily drug intake of 3.2 medications (IQR: 1.25–4.00).

Baseline characteristics of the patients and the details of their drug therapies are summarized in Table 1.

### 3.2. Evaluation of Suggestions Shared by the Clinical Pharmacist with Clinicians

During the observation period, the clinical pharmacist provided a total of 138 recommendations for 57 patients, with a median of 2 suggestions for each patient (range 1–3).

The most frequently shared suggestion clusters were “Medication Errors” (D, 45%), “Drug Supply” (E, 26%), and “Pharmaceutical Form Optimization” (A, 18%). When examining individual recommendation types, the most common suggestions provided by the pharmacist were related to “drug–drug interactions” (D1, 23%), followed by “compounding or purchasing medications not included in local formulary” (E2, 17%).

### 3.3. Evaluation of Suggestions Accepted by Clinicians

The analysis of shared pharmaceutical recommendations revealed a total of 138 suggestions, of which 42% (*n* = 58) were accepted and 58% (*n* = 80) were not. The most frequently shared category was medication errors (D) (45%, *n* = 62), particularly related to drug–drug interactions (D1, 23%), medication review (D5, 10%), and food–drug interactions (D2, 6%). Among these, 32% (*n* = 20) were accepted. Drug supply (E) was the second most common cluster (26%, *n* = 36), mainly driven by compounding or purchasing extra-formulary medications (E2, 17%). Among the “E cluster”, 42% (*n* = 15) of shared suggestions were accepted. Pharmaceutical form optimization (A) accounted for 18% (*n* = 25) of the suggestions, particularly formulation changes for safer handling (A2, 8%) and advice on handling (A3, 9%), with an overall acceptance of 64% (*n* = 16). Conversely, posology optimization (B), drug change (C), and handling and administration (F) were less often shared. Within the same suggestion categories (A, B, C, D, E, or F), the suggestions most frequently accepted by clinicians involved handling and administration (F), pharmaceutical form optimization (A), drug change (C), and drug supply (E).

These findings highlight the key areas where interventions were most and least likely to be implemented, suggesting a higher feasibility for drug supply adjustments and formulation changes, while modifications related to medication errors required further assessment before acceptance. See Table 2 for further information.

During the observation period, the clinical pharmacist shared 138 suggestions for 57 patients, with a median of 2 suggestions for each patient (range 1–3).

### 3.4. Analysis of Time to Intervention and Acceptance Rate Correlation

During the 13 weeks of the study, a comprehensive analysis of the degree of acceptance of shared recommendations was conducted.

Throughout this period, the median number of recommendations sent per week was 12.

The weekly trend of shared/accepted recommendations provides an indication of how the acceptance rate correlates with the consolidation of the clinical pharmacist’s activity.

**Figure 3:**
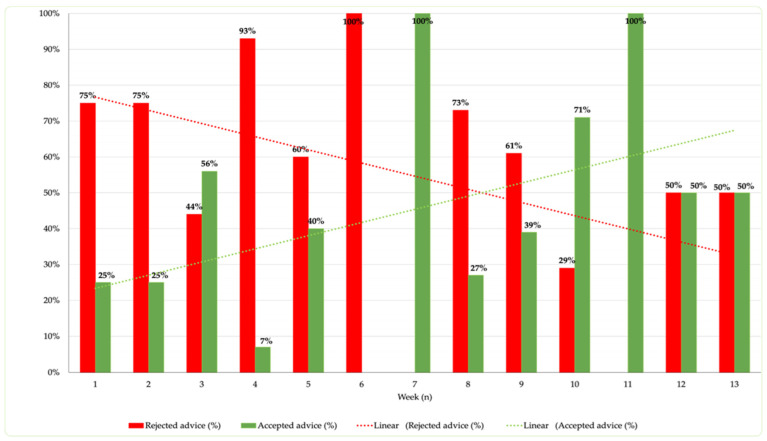
Chart of suggestions provided weekly to the Pediatric Neurology and Neurophysiology Unit.

As reported in Figure 3, indeed, it is noted that, as the weeks of project implementation progress, the proportion of accepted recommendations per week increases. Except for week 6, the trend demonstrates an increasingly higher frequency of accepted recommendations, with two peak values (100%) reached in weeks 7 and 11. The introduction of the clinical pharmacist in the weekly departmental meetings has facilitated an increase in trust from the department clinicians, resulting in a percentage increase in accepted recommendations. This observation is supported by the increasing trend, positively correlated with the weeks of observation (R^2^ = 0.59).

The structuring of weekly commitment and the implementation of discussion sessions, such as clinical case review meetings, have contributed to improving the acceptance of recommendations at the Pediatric Neurology and Neurophysiology Unit.

## 4. Discussion

The setting selected in this study involves pediatric patients with epilepsy, representing an important area of study and requiring carefully monitored antiepileptic therapy to ensure both treatment efficacy and patient safety.

The concurrent use of multiple antiepileptic drugs poses complex challenges related to pharmacological interactions, potentially affecting treatment response and tolerability. Continuous treatment is crucial, as non-adherence can lead to recurrent seizures, significantly impacting the patient’s quality of life.

An essential aspect is the careful evaluation of drugs used in pediatric patients, with particular attention to the use of drugs not specifically approved for this population or not intended for the treatment of epileptic seizures. This carries the risk of unforeseen side effects and requires careful assessment by the clinical pharmacist.

In our study, the impact of the clinical pharmacist’s intervention was analyzed by observing the acceptance level of shared suggestions with physicians over a 13-week period. During this time, a total of 138 suggestions were shared with the analyzed patients, of which 42% were accepted by the prescribers. Although this outcome serves more as a methodological indicator than an effectiveness measure, it provided valuable insights into the actual perception of the pharmacist’s activities.

The “traditional” support of the pharmacist in medication reviews involves identifying potential DRPs but requires time and the establishment of a “culture” to achieve a satisfactory acceptance level [18].

For this reason, we clustered the types of recommendations provided by the pharmacist to analyze areas where the professional’s opinion was well accepted versus areas requiring sensitization among the healthcare providers receiving the suggestions.

The most shared suggestion was about medication errors (45%), which remains the responsibility of clinical pharmacists, as highlighted by the study Zhang et al. [19]. However, the acceptance rate of type D suggestions was only 32%. Other areas had proven more suitabile to changes, like drug supply adjustments and formulation changes.

The longitudinal analysis of suggestions provided over 13 weeks revealed significant variability in acceptance and rejection rates. The acceptance of suggestions varied throughout the study, driven by increased collaboration between pharmacists and clinicians through weekly meetings and the clinical pharmacist’s growing understanding of the patient cohort. These fluctuations could be attributed to several factors, including the hospital staff’s increasing familiarity with the clinical pharmacist, the complexity of cases treated, and interprofessional communication [20].

It is noteworthy that some weeks recorded a 100% acceptance rate of recommendations, suggesting a better understanding and trust in the role of the clinical pharmacist. On the other hand, weeks with higher rejection rates may reflect initial resistance to change or inadequate communication.

### 4.1. Strengths and Weaknesses (Study Limitations)

The study, while providing important results, presents some limitations.

Firstly, the only parameter used to evaluate the impact of the clinical pharmacist was the number of suggestions accepted or rejected by physicians. However, this endpoint may not fully capture the effectiveness of the pharmacist’s role, as it does not account for other clinically relevant variables, such as the effects of therapeutic modifications on patients, drug-related problems, hospitalization length, and other outcome indicators. Also, the absence of a control group or a historical comparator does not allow us to attribute the improvements observed exclusively to the involvement of the clinical pharmacist.

A second aspect to consider is the relatively short observation period. This, combined with a ward organization that included the closure of scheduled admissions between weeks 10 and 13, could have influenced the data and the overall representativeness of the results.

Additionally, the lack of specific training for the ward staff may have impacted their perception and collaboration with the clinical pharmacist. The absence of dedicated resources for continuous pharmacist involvement is another obstacle, limiting regular interventions in the ward.

Expanding the activities with more continuous participation in specialist meetings, as well as involvement in ward rounds and all medication review phases, not only for patients with scheduled admissions, will be crucial to verify the definitive impact of the clinical pharmacist in an Italian pediatric setting.

### 4.2. Further Research

An aspect to examine is the potential implementation of medication reconciliation during care transitions, namely immediately after admission to the ward and upon transfers to other operating units or home. This initiative aims to optimize the management of pharmacological therapy throughout the entire care pathway, contributing to reducing potential errors or inefficiencies.

A necessary step, once all information on the ongoing project has been collected, is to promote the clinical pharmacist figure in all pediatric settings, particularly those where polypharmacotherapy is frequent. This sharing would allow for the analysis of the clinical pharmacist’s impact on the pediatric patient from a 360-degree perspective, enriching the evaluation framework.

To promote even closer collaboration with the medical team, it could be considered to involve the clinical pharmacist more continuously in ward activities through participation in daily ward meetings and patient visits. This would ensure a constant presence and facilitate more effective synergy among the professional figures involved.

Finally, it would be useful to expand consultation activities, extending support not only to patients undergoing pre-admission but also to those admitted in emergency. This approach aims to improve medication management and the quality of care provided throughout all stages of the care pathway.

These prospects represent potentially useful directions for enriching the project and deepening the evaluation of the clinical pharmacist’s effectiveness within the pediatric hospital setting.

Moreover, the 13-week observation period may be insufficient to capture long-term trends or outcomes. Future research should consider extended follow-up to better assess the sustainability and impact of pharmacist interventions.

## 5. Conclusions

In the context of the Pediatric Neurology and Neurophysiology Unit, the introduction of the clinical pharmacist has proven to be a valuable element in patient care.

Our research has highlighted how this intervention had a significant impact on increasing adherence to recommendations over the 13-week study period.

The clinical pharmacist has established effective communication within the multidisciplinary team, playing a crucial role in monitoring therapeutic appropriateness and in training healthcare staff and caregivers to optimize and make drug administration safer. This research project has clearly demonstrated how involving a clinical pharmacist in a multidisciplinary clinical setting is a valuable resource for managing complex patients.

While acceptance of shared recommendations still has room for improvement, it is fascinating to observe how this process has sparked constructive debate and promoted increased communication and collaboration between clinical pharmacists and physicians, with the latter becoming more open to collaboration and sharing.

Through research projects like this and by implementing tailored training opportunities in clinical pharmacy from university education onwards, it will be possible to institutionalize this professional role in Italy, enhancing patient care and fostering an important and innovative professionalism for pharmacists.

## Figures and Tables

**Table 1:** Baseline patients’ characteristics and drug therapy details.

Variables	
Gender, *n* (%)	**Total (N = 57)**
Female	27 (47%)
Male	30 (53%)
Age, *n* (%)	
<1 year	11 (19%)
1–5 years	19 (33%)
6–12 years	17 (30%)
13–18 years	10 (18%)
Age, median years (IQR)	3.0 (1.00–10.25)
Drug Therapy, median (IQR)	3.2 (1.25–4.00)
Polypharmacy, *n* (%)	
drugs taken > 5	9 (16%)
drugs taken > 10	2 (4%)
Prescribed drugs by ATC * code, *n* (%)	**Total (N = 160)**
N—nervous system	111 (69%)
A—alimentary tract and metabolism	17 (11%)
R—respiratory system	8 (5%)
Other ATC codes	16 (10%)
Supplements	8 (5%)

* Anatomical Therapeutic Chemical (ATC) classification.

**Table 2:** Accepted vs. not accepted suggestions by cluster.

Not Accepted, *n* (%) ^c^	Accepted, *n* (%) ^b^	Total, *n* (%) ^a^	Cluster of Suggestions
9 (36%)	16 (64%)	25 (18%)	A—Pharmaceutical Form Optimization
1 (100%)	-	1 (1%)	A1—formulation change for better compliance
6 (55%)	5 (45%)	11 (8%)	A2—formulation change for safer handling
2 (15%)	11 (85%)	13 (9%)	A3—advice on handling
5 (71%)	2 (29%)	7 (5%)	B—Posology Optimization
2 (100%)	-	2 (1%)	B1—time of administration
-	2 (100%)	2 (1%)	B2—meals-related administration time
3 (100%)	-	3 (2%)	B3—posology change (dosage, time)
1 (50%)	1 (50%)	2 (1%)	C—Drug Change
1 (50%)	1 (50%)	2 (1%)	C1—analog in the hospital formulary
42 (68%)	20 (32%)	62 (45%)	D—Medication Errors
28 (88%)	4 (12%)	32 (23%)	D1—drug–drug interactions
4 (50%)	4 (50%)	8 (6%)	D2—food–drug interactions
-	1 (100%)	1 (1%)	D3—off-label use
1 (100%)	-	1 (1%)	D4—therapy discontinuation
8 (57%)	6 (43%)	14 (10%)	D5—medication review
1 (17%)	5 (83%)	6 (4%)	D6—adverse drug reaction
21 (58%)	15 (42%)	36 (26%)	E—Drug Supply
4 (33%)	8 (67%)	12 (9%)	E1—new drug to be listed in the internal formulary
17 (71%)	7 (29%)	24 (17%)	E2—compounding or purchasing extra-formulary medications
2 (33%)	4 (67%)	6 (4%)	F—handling and administration
2 (33%)	4 (67%)	6 (4%)	F1—easier drug delivery (mixed with food, diluted in water)
**80 (58%)**	**58 (42%)**	**138 (100%)**	**Total**

^a^ (*n*/138) %; ^b,c^ (*n*/total per table row) %.

## Data Availability

Due to privacy and ethical reasons, data are available from the authors upon request to the corresponding author.
